# Managing the Lower Lip-Sucking Habit With a Lip Bumper Appliance: A Pediatric Case Study

**DOI:** 10.7759/cureus.65575

**Published:** 2024-07-28

**Authors:** Yadnya U Kawale, Himani Parakh, Nilima R Thosar, Aakriti Chandra

**Affiliations:** 1 Dentistry, Sharad Pawar Dental College and Hospital, Datta Meghe Institute of Higher Education and Research Centre, Wardha, IND; 2 Pediatric and Preventive Dentistry, Sharad Pawar Dental College and Hospital, Datta Meghe Institute of Higher Education and Research Centre, Wardha, IND

**Keywords:** crowding, functional appliance, parafunctional, oral habit, lip sucking

## Abstract

Oral habits in children are of prime concern to a pediatric dentist as the child is in an active growth phase, and it may alter the development of the orofacial area. One of the major etiological factors in the development of malocclusion and other adverse effects on orofacial structures is oral habits during and after preschool age. The habit of sucking one's lower lip is rarely examined; it appears that practitioners tend to associate it with less adverse clinical consequences. It is important to identify harmful oral habits and eliminate them as early as possible. This case report focuses on the diagnosis of the lip-sucking habit, understanding its etiology, and creating a treatment plan that will prevent unfavorable consequences. A lip bumper is an easy-to-fabricate, easy-to-place, and easily tolerated intra-oral appliance. The successful use of lip bumper in this case adds to the evidence that when used as an early intervention the habit can be intercepted appropriately.

## Introduction

Children frequently acquire oral behaviors that could damage dental occlusion and its supporting structures permanently or just temporarily. These are very complex behaviors that are ingrained patterns of contraction of the muscles. Infantile periods are characterized by repetitive behaviors, the majority of which begin and end on their own [1]. According to Morris and Bohanna (1969), the following classifications were used to categorize oral habits: pressure, non-pressure, and biting. Pressure habits, which include thumb sucking, tongue thrusting, finger sucking, and lip sucking exert direct force on the teeth or the tissues that support them. Non-pressure habits, like mouth breathing, do not exert direct pressure on the teeth or the tissues that support them. Biting habits include nail biting and lip biting. Certain malocclusions, such as Angle's class II malocclusion, increased overjet, open bite, posterior crossbite, and small maxillary width, are known to be brought on by oral habits [2].

Numerous habits include tongue thrusting, lip sucking, mouth breathing, bruxism, nail biting, and thumb sucking. Throughout infancy and the first few years of childhood, an oral habit is natural, and if prolonged after three years of age, it is considered abnormal [3]. There are two major types of sucking that are easily identifiable: nutritive sucking and non-nutritive sucking [4]. Nutritive sucking is a type of sucking that gives humans the necessary nutrients they need, whereas non-nutritive sucking gives the child a sense of warmth, safety, and belonging; however, it does not give them any nutrition [5]. Non-nutritive sucking is linked to malocclusion in the primary teeth and exerts a significant stress on the dentition [6]. The buccinator, mentalis, and orbicularis oris muscles provide additional oral forces, which are counterbalanced by the tongue's opposing forces [7]. According to a study done in the central region of Kerela, the altogether widespread presence of oral habits was 72.7% whereas the prevalence of lip-sucking habits alone was 4.5% [8]. Lip sucking is the technique of continuously holding the lower lip between the upper and lower anterior teeth. The lower lip is most frequently affected; it is turned inward and is compressed on the inside [9]. Lip sucking is categorized as a form of non-nutritive sucking.

Crowding and deep bite are the two most commonly seen malocclusion associated with the lip-sucking habit. Crowding is considered to be a malocclusion that never gets better on its own, instead it becomes worse with time. In the following two dental phases, if malocclusion is present in deciduous teeth, it will continue to get worse [10]. One of the most common conditions that harm malocclusion from the perspective of future dental and masticatory devices is deep bite [11]. Lip sucking is very common among children, and it can be due to stress. Overbite also causes lip-sucking habits. Over time, excessive lip-sucking habits might result in dry, chapped, or irritated lips. Lip sucking is not self-correcting, and when a deep bite is included with it, it may become even more destructive. 

## Case presentation

**Figure 1:**
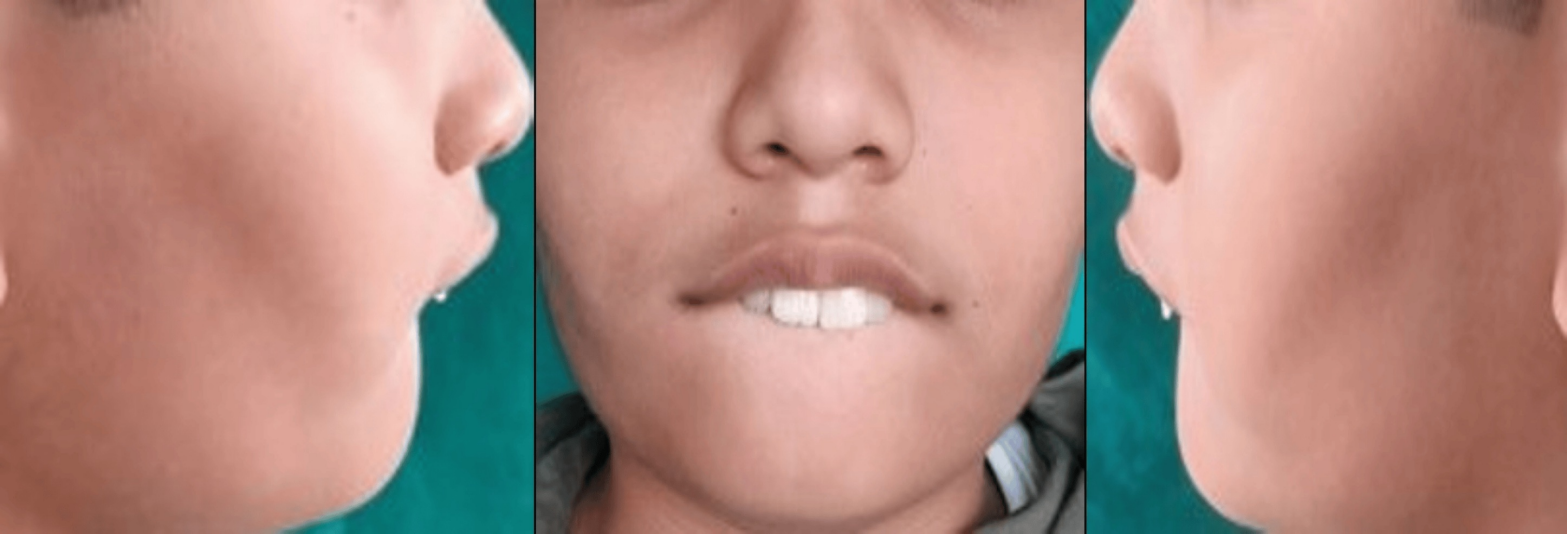
Photographs of the patient while performing the habit

A 12-year-old male came to the Department of Pediatric and Preventive Dentistry accompanied by his parents with a complaint of pain in the upper left back region of his jaw for seven days. The pain was sharp shooting in nature, aggravated on drinking cold water, and was relieved on its own with no significant history of night pain. Further examination and consultation with parents disclosed the child’s habit of frequently sucking his lower lip for five to six years. The patient had a known case of epilepsy and had been under treatment for one year; since then no episodes of epilepsy had been experienced by the patient. He underwent dental restorative treatment at a private clinic two to three months back, and there were no significant complications present during the treatment. In the past, none of his family members were diagnosed with a similar condition. No relevant drug history was recorded. On extra oral examination, the face was grossly symmetrical with a straight facial profile. Bilateral synchronous temporomandibular joint movements with no clicking or popping sound were heard. The patient also showed an improper lip seal while performing the habit (Figure 1).

**Figure 2:**
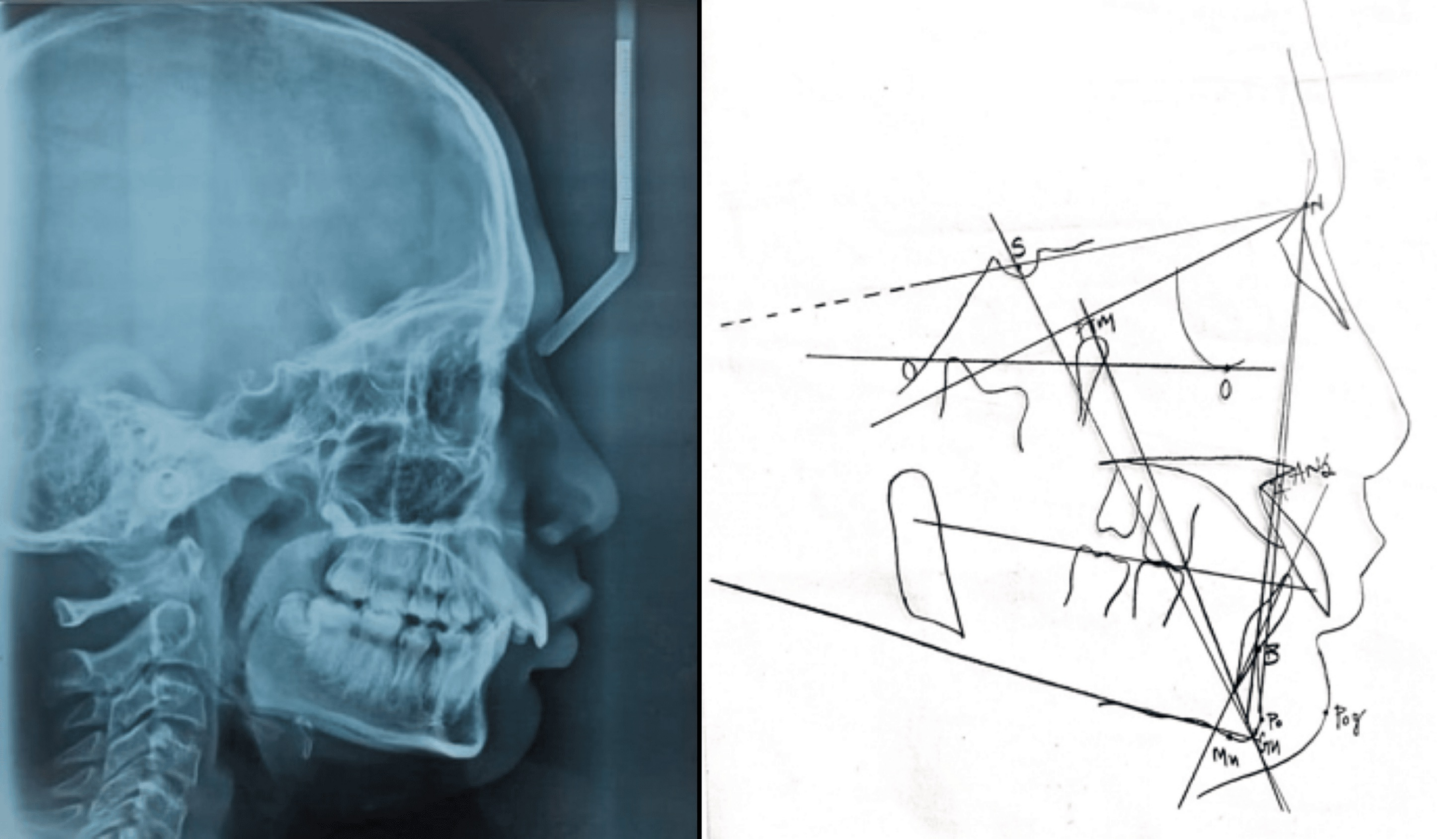
Lateral cephalogram and cephalometric analysis Figure created by authors (right panel)

On intraoral examination, a class I molar relationship was seen. In addition, enamel chipping was seen with #16 with a history of food lodgement. Oral prophylaxis and pit and fissure sealant were done with #36 and #46. Composite restoration was done with #16 followed by a habit-breaking appliance. In this case, the habit was identified after observing potentially competent lips, deepened chin, deep bite, proclination, persistent lip wetting, and chapped lips. A lateral cephalogram radiograph was advised for investigation (Figure 2). Cephalometric analysis revealed a facial depth angle of 85°. A labial inclination of maxillary teeth was seen (A1-Po angle = 24° and A1-Po distance = 5 mm), whereas the mandibular inclination of teeth was (B1-Po distance = 15 mm). Lower anterior facial height ANS-Gn was 56 mm. From the left and right lower molars to the pterygoid vertical (PTV) line the distance was 30 mm and 28 mm, respectively. From the upper lip and lower lip to the esthetic plane, the distance was -2.5 and -3, respectively. The maxillary length ANS-PNS was 45 mm, and the mandibular length Co-Gn was measured to be 108 mm.

**Figure 3:**
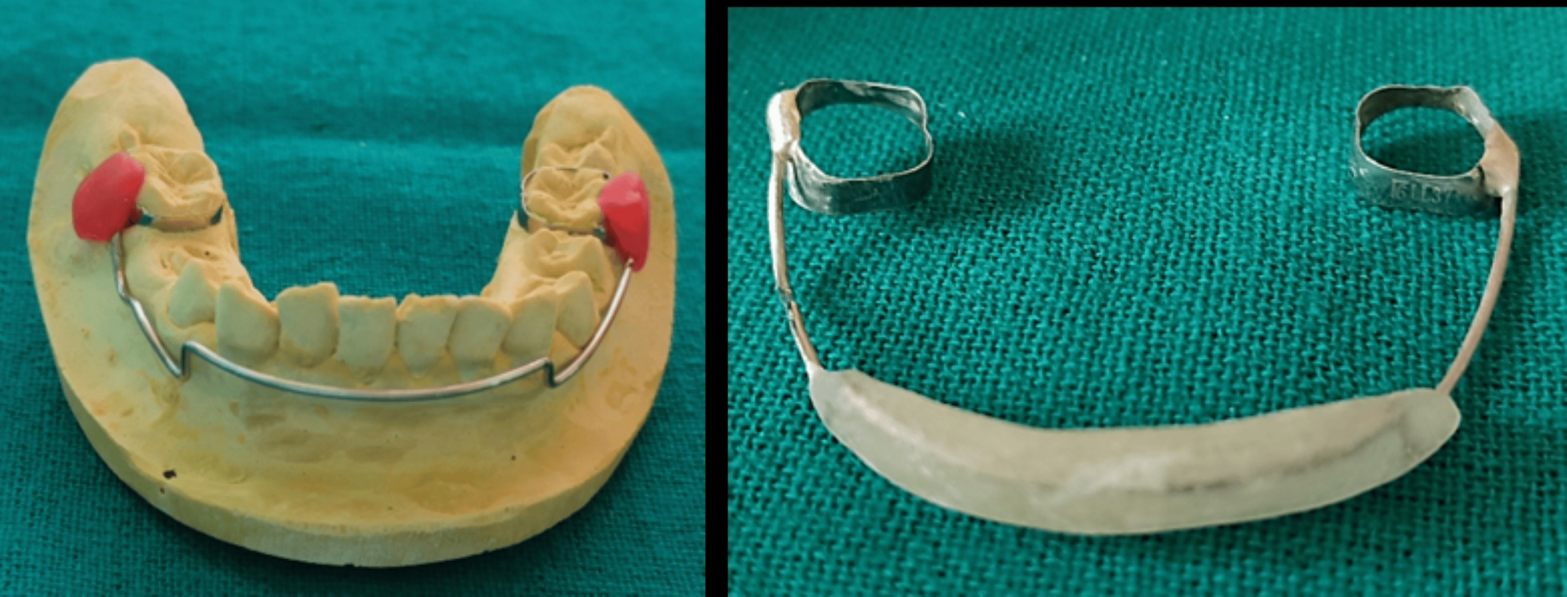
Fabrication of the wire and acrylic component of the lip bumper appliance

Hence, considering all the clinical and radiographic findings, the patient was diagnosed with a lip-sucking habit. A lip bumper was designed to overcome the habit. Alternative treatment options for lower lip sucking include oral screen and maxillary appliance. Treatment in this case aimed to reduce the deep bite and eliminate the habit of lip sucking to enhance facial function and appearance. The establishment of the diagnostic model was followed by the working model, which helped in designing and constructing the appliance. For the fabrication of the appliance, pre-formed bands (3M Unitek) were selected for lower permanent molars #36 and #46. Maxillary and mandibular impressions were made using alginate impressions (Dentsply Sirona, Germany). For the construction of the appliance, a 19-gauge stainless steel wire (Konark, India) was used. The anterior part of the wire component was kept 4-5 mm away from the incisors. Fabrication of the acrylic part was done with cold cure acrylic material (clear acrylic DPI-RR self-cure). Further, soldering of the band material and wire component was done (Figure 3).

**Figure 4:**
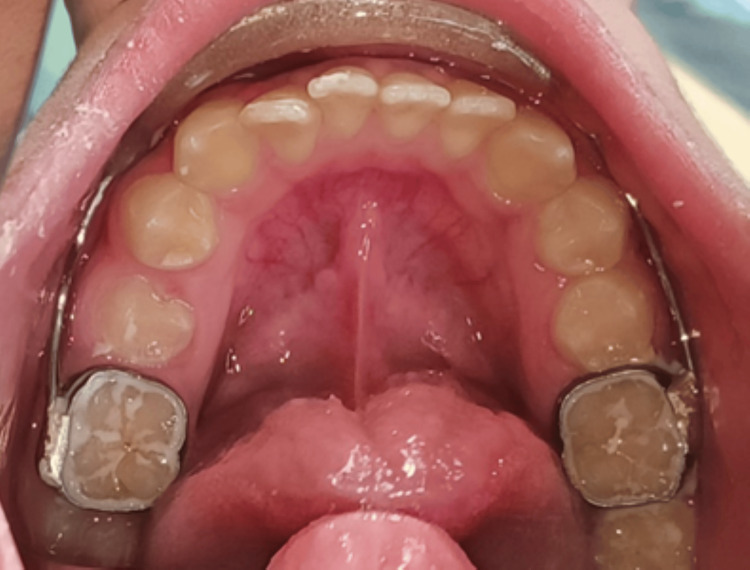
Lip bumper appliance cemented intraorally

**Figure 5:**
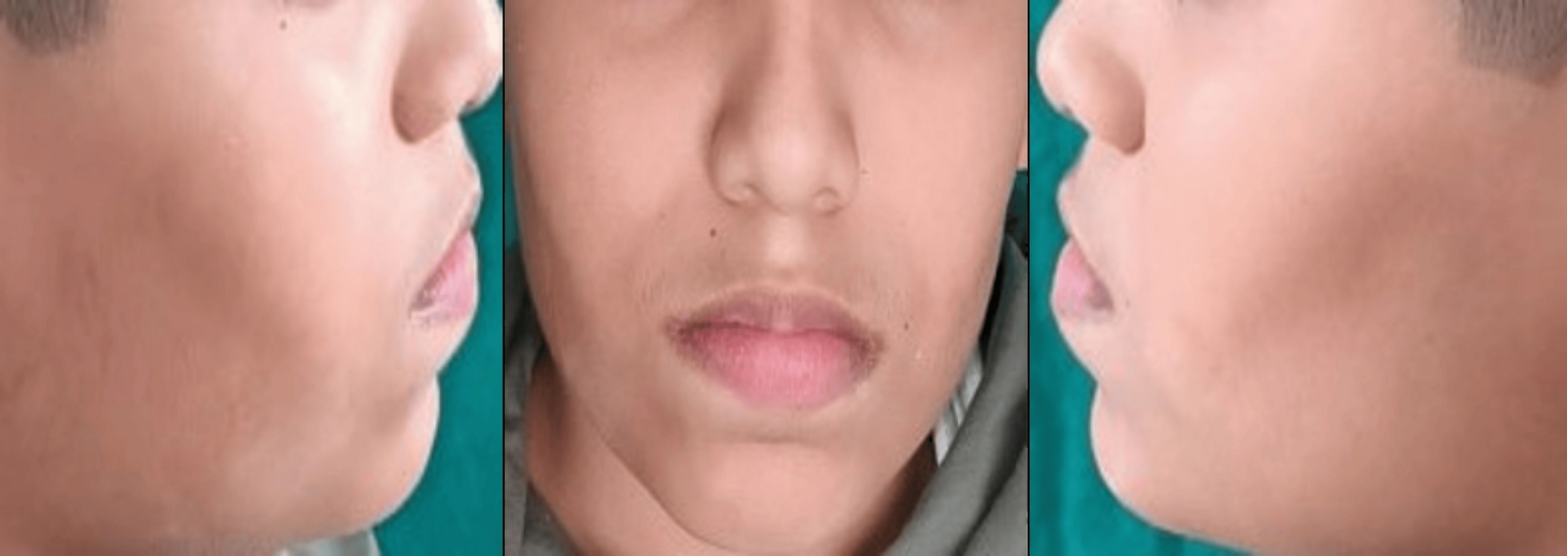
Immediate post-op photographs

**Figure 6:**
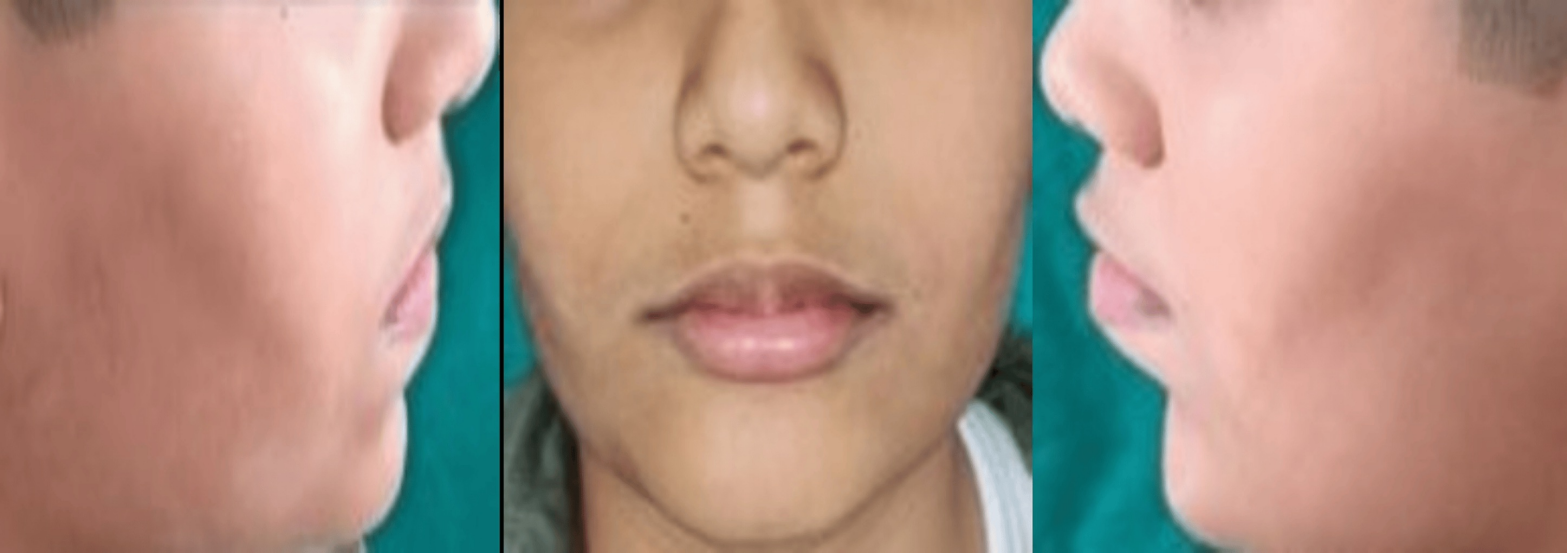
Six-month follow-up photographs

The appliance was tried for better patient compliance. Cementation of the lip bumper using GIC Type I luting cement and delivery of the appliance was done (Figure 4). Immediate post-operative photographs were recorded (Figure 5). Both the patient and the parents cooperated well throughout the procedure. Follow-ups were taken at one, three, and six months, and at the end of six months, cessation of the habit was observed (Figure 6).

## Discussion

This may be the case because nine is considered a preadolescent age, during which children commonly experience physiological, psychological, emotional, and social changes. A child's dentition may become disfigured as a result of the mixed dentition era, which begins at age six [12]. Any prolonged disruption of the balance in the muscle activity can cause parafunctional oral habits like lip sucking, nail biting, or tongue thrusting that might disturb the equilibrium, begin a morphologic shift in the normal alignment of the teeth and supporting bone, and lead to a malocclusion [13]. Usually, a bottle with an artificial nipple is used to feed a baby when being looked after by a babysitter. The sucking reflex may not develop as a result of this circumstance. Anxiety and stress from this may result in lip-sucking habits. If this response is not given enough time to mature, children's sucking habits may persist into later childhood and lead to dental malocclusion. For this reason, it is important to allow the sucking reflex to develop fully through appropriate breastfeeding [14]. If children develop a habit of lip sucking daily, it needs to be recognized and treated with appropriate preventive measures to break this habit. When they are nervous or anxious, children generally suck their lips. The child may not be at risk for dental development from this type of lip sucking because they only do it occasionally. Certain clinical indicators, such as reddish or chapped lips, persistent lip wetting, tooth prints on the lip, deepened chin sulcus, protrusion of the upper front teeth, or retrusion of the lower teeth can be used as a measure to identify this habit [15]. Various clinical presentations seen are lip incompetence, incisor protrusion, posterior crossbite, and anterior open bite. Some of the common adverse effects of long-term oral habits include distal step molar relation. Depending on the child's unique bone and dental structure, established habits, and other factors, the severity of these disturbances varies. It is considered an indication of mental instability and discomfort when preschool-aged children develop these parafunctional habits [16].

Numerous studies have shown that lip bumpers can improve adverse oral habits, help regain lower arch space, and manage molar anchorage [17]. A lip bumper is a special myofunctional appliance that helps eliminate the lower lip-sucking habit. The patient is encouraged to gradually decrease and overcome the habit in addition to being made to understand its negative effects. The lip bumper prevents the mentalis muscles from contracting excessively, which stops abnormal stresses from acting on the incisors [18]. An acrylic shield is placed over the anterior wire to strengthen the lip and cheek muscles' adaption. The lower lip bumper is a simple-to-use appliance for correcting habits, and young patients usually accept it well [17]. But in some cases, lip sucking can be caused by muscular imbalance, which typically leads to incompliant lips and increases the chance that a child might suck the lip [3].

Lip sucking is a harmful oral habit that interferes with appropriate lip occlusion and growth. The frequency of lip sucking varies from 2.2% to 4.8% between the ages of infancy and six. It is a rather uncommon practice [19]. Lip sucking can aid in the development of the class II skeletal relationship. A skeletal maxillary protrusion brought on by an inherited interference or congenitally absent teeth, allergic rhinitis, mouth breathing, or as a substitute for thumb sucking can also cause lip sucking [20]. As the appliance was installed, the patient was informed that it was supposed to be worn for five to six months before the habit could no longer be felt.

## Conclusions

The use of lip bumper appliance therapy in pediatric patients with lip-sucking habits has proven to be an effective non-invasive method as presented in this case report. At the end of the treatment, there was a significant reduction in the active habit of the patient and a marked change was observed in the oral musculature. Patient compliance and regular follow-up were as crucial as the interceptive treatment provided. This case provides an understanding that early interception in childhood is beneficial.
